# Development and validation of the Upstream Social Interaction Risk Scale (U-SIRS-13): a scale to assess threats to social connectedness among older adults

**DOI:** 10.3389/fpubh.2024.1454847

**Published:** 2024-09-16

**Authors:** Matthew Lee Smith, Matthew E. Barrett

**Affiliations:** ^1^Center for Community Health and Aging, Texas A&M University, College Station, TX, United States; ^2^Department of Health Behavior, School of Public Health, Texas A&M University, College Station, TX, United States

**Keywords:** social disconnectedness, loneliness, social isolation, scale validation, older adult

## Abstract

**Background:**

Social interactions are essential to social connectedness among older adults. While many scales have been developed to measure various aspects of social connectedness, most are narrow in scope, which may not be optimally encompassing, practical, or relevant for use with older adults across clinical and community settings. Efforts are needed to create more sensitive scales that can identify “upstream risk,” which may facilitate timey referral and/or intervention.

**Objective:**

The purposes of this study were to: (1) develop and validate a brief scale to measure threats to social connectedness among older adults in the context of their social interactions; and (2) offer practical scoring and implementation recommendations for utilization in research and practice contexts.

**Methods:**

A sequential process was used to develop the initial instrument used in this study, which was then methodologically reduced to create a brief 13-item scale. Relevant, existing scales and measures were identified and compiled, which were then critically assessed by a combination of research and practice experts to optimize the pool of relevant items that assess threats to social connectedness while reducing potential redundancies. Then, a national sample of 4,082 older adults ages 60 years and older completed a web-based questionnaire containing the initial 36 items about social connection. Several data analysis methods were applied to assess the underlying dimensionality of the data and construct measures of different factors related to risk, including item response theory (IRT) modeling, clustering techniques, and structural equation modeling (SEM).

**Results:**

IRT modeling reduced the initial 36 items to create the 13-item Upstream Social Interaction Risk Scale (U-SIRS-13) with strong model fit. The dimensionality assessment using different clustering algorithms supported a 2-factor solution to classify risk. The SEM predicting highest risk items fit exceptionally well (RMSEA = 0.048; CFI = 0.954). For the 13-item scale, theta scores generated from IRT were strongly correlated with the summed count of items binarily identifying risk (*r* = 0.896, *p* < 0.001), thus supporting the use of practical scoring techniques for research and practice (Cronbach’s alpha = 0.80).

**Conclusion:**

The U-SIRS-13 is a multidimensional scale with strong face, content, and construct validity. Findings support its practical utility to identify threats to social connectedness among older adults posed by limited physical opportunities for social interactions and lacking emotional fulfillment from social interactions.

## 1 Introduction

Social interactions are essential to social connectedness among older adults. Social connectedness refers the structural (e.g., network size and composition, partner status), functional (e.g., perceived social support, loneliness), and quality (e.g., relationship quality or strain) aspects of an individual’s social relationships (1, 2). Taken together, social connectedness can be thought as an umbrella term to encompass social isolation (objectively having limited contact with others) and loneliness (subjective feeling of being alone) (2, 3). An estimated 25% of older adults are thought to be socially isolated (3) and over 40% are thought to be lonely (2, 4, 5), which can be a staggering figure considering these concepts are interrelated but do not necessarily overlap completely (2, 3). Older adults who are socially connected have a reduced risk of all-cause mortality (6, 7), but those experiencing social isolation and loneliness are at an increased risk of diminished physical (e.g., heart disease, stroke) (8, 9) and mental health (e.g., depression, anxiety) (10), cognitive impairment (11, 12), and risky behaviors (e.g., substance use, sedentary behavior) (13–15). As such, it is imperative that we identify threats to social connectedness early through routine screening across community sectors and facilitate meaningful social interactions among and between older adults.

Many validated scales have been developed to measure various aspects of social connectedness (3, 16). These scales are frequently used in the research context and are increasingly used to identify baseline risk (often for service recommendations or referrals) and evaluate the effectiveness of clinical and community-based interventions (3, 17). Each of these scales were purposively developed to measure a specific aspect or trait of social connectedness; therefore, they may be seen as assessing only one component within a larger set of interrelated risks (18). This can create operationalization-related issues and implications for social care because, while social disconnectedness is a multi-faceted problem, the singular outcome selected for use is frequently treated as the sole indicator of how an older adult perceives their overall social wellbeing. As such, in research and practice, administering a combination of multiple scales may be necessary to provide a more comprehensive view of risk or threats to social connectedness, which may introduce undue data collection burdens.

The scales commonly used in research and practice have helped the field define and quantify the prevalence of risk within the older adult population (3, 18). However, many of these scales may not be optimally encompassing, practical, or relevant for use with older adults across clinical and community settings. These scales are valuable to identify dimensions of risk, but when used independently, they may not adequately capture the complexity of all existing risk or guide intervention opportunities. Based on their generation of ample evidence, these scales provide a solid foundation on which to create new, contextually appropriate scales to assess risk among older adults. Further, given the overlap of concepts and items across existing scales, it may be practical to examine how these scales work together to identify risk and if underlying concepts can be captured with a single compilation of measures. Building upon the legacy and strength of existing scales, especially pervasive and commonly use scales such as the UCLA Loneliness Scale (19–21), it may be possible to create a more encompassing and sensitive scale that can identify “upstream risk” and detect the maximum amount of threat to social connectedness. Intentionally using more generous risk scoring algorithms may enable researchers and practitioners to identify older adults upstream (i.e., with emerging threats to social connection), which may allow for timely referral and/or intervention. Therefore, the purposes of this study were to: (1) develop and validate a brief scale to measure threats to social connectedness among older adults in the context of their social interactions; and (2) offer practical scoring recommendations for utilization in research and practice settings. Social interactions were conceptualized in terms of the physical opportunities to interact with others (e.g., having a social network, ability to find companionship, going to gathering places) and the emotional fulfillment resulting from those interactions (e.g., satisfaction, content, longing). In this context, limited physical opportunities to interact and/or emotional fulfillment from interactions may place an older adult at risk for social disconnectedness. The availably of a brief, sensitive, and easy-to-administer scale that more broadly assesses threats to social connectedness can complement the inventory of existing measures and assist researchers and practitioners to efficiently identify multi-faceted risk among older adults.

## 2 Methods

### 2.1 Preliminary instrument development

A sequential process was used to develop the initial instrument used in this study, which was then methodologically reduced to create a brief 13-item scale. The overall intent was to identify a wide range of existing, validated scales and items capable documenting the complexity of social connectedness as an encompassing concept, then employ a series of statistical analyses to identify the most parsimonious set of items to measure threats to social connectedness. The goal of developing a parsimonious brief measure from this process was to increase practical administration in research and practice settings.

First, 13 validated scales were identified from the published literature based their relevance to various social connectedness aspects, presence in the published literature, and use in research and practice with older adults. These scales included the Berkman-Syme Social Network Index (22), Brief Sense of Community Scale (23), Campaign to End Loneliness Measurement Tool (24), Connect2Affect Assessment (25), Connor-Davidson Resilience Scale (26), Cornwell Perceived Isolation Scale (27), de Jong Gierveld Loneliness Scale (28), Duke Social Support Index (29), Life Space Questionnaire (30), Lubben Social Network Scale (31), Patient Health Questionnaire-2 (32), Steptoe Social Isolation Index (33), and Revised UCLA Loneliness Scale (19, 20). During this process, the goal was to identify as many relevant scales and measures as possible that could be associated with social interactions among older adults.

Second, these scales were critically reviewed for content and overlapping concepts to optimize the potential universe of relevant items to assess threats to social connectedness while reducing potential redundancies. This process was undertaken by a combination of research and practice experts (*n* = 3), who ranked each item within each identified measure in terms of its relevance to social connectedness. When consensus was not reached about whether to include an item, the experts elected to retain the item to be more inclusive at this stage.

Third, an expanded panel of experts (*n* = 19) was engaged to review the collection of identified items, evaluate their appropriateness, recommend items to fill concept gaps, and/or offer alternative wording and response choices for presented items. Experts were selected from within the researchers’ professional network based on their content expertise and experience engaging older adults in screening and service delivery. This expanded expert panel included a diversified group of clinicians, professionals, and community members. More specifically, experts represented the disciplines of public health, gerontology, medicine, nursing, social work, psychology, physical therapy, and health education. Further, experts included representatives of Area Agencies on Aging, community health workers, caregivers of older adults, and community-dwelling older adults. This process was not a formal consensus-building effort in that experts were not convened together or asked to agree upon the initial instrument to be tested. Rather, experts were asked to provide their feedback individually (i.e., in written or verbal format), and their responses were shared with other experts when appropriate.

Recommendations were assessed, accepted, and incorporated into the initial instrument. A total of 36 items were included to assess threats to social connectedness, which were included as part of a larger survey of older adults. Finally, the instrument was piloted by older adults (*n* = 5) who provided feedback on the items in terms of comprehension, readability, and appropriateness.

### 2.2 Measures

Table 1 presents the initial 36 items used to assess threats to social connectedness along with their source(s) of origin and response categories. The compiled items originated from 12 sources. All items were close-ended and used one of five different sets of response categories (e.g., yes/no; none/one/two or more; none of the time/some of the time/often). To capture the maximum amount of reported risk and create more uniformity across items, responses for each item were recoded to a binary state that indicates “risk” and “no risk.” This was accomplished by scoring response choices indicative of the absence of risk as 0 and all other response choices as 1 (see Table 1). Recoding each item accounted for differing response categories, while retaining the sensitivity of the original item format (e.g., an affirmation of an item to any degree was taken as an affirmation) and allowed for the detection of upstream risk.

**Table 1:** Initial 36 items used to develop the U-SIRS-13.

Items	Scale of origin	Response categories	Included in U-SIRS-13	Intended concept measured in scale*	Aspect of social connection measured*
I avoid socializing because it is hard to understand conversations, especially when there is background noise	Connect2Affect	Yes, No	X	Self-Restricted Activity	N/A
I am satisfied with the relationships I have with my family	Duke Social Support Index (DSSI), Campaign to End Loneliness (CEL)	Yes, No	X	Social Connection, Loneliness	Structure, Function, Quality
I am satisfied with the relationships I have with my friends	DSSI, CEL	Yes, No	X	Social Connection, Loneliness	Structure, Function, Quality
I have as much contact as I would like with people I feel close to and who I can trust and confide	DSSI	Yes, No	X	Social Connection	Structure, Function, Quality
There are enough people I feel close to and could call for help	de Jong Loneliness, CEL	Yes, No	X	Social Connection, Social Isolation, Loneliness	Structure, Function, Quality
I am content with my friendships and relationships	Berkman-Syme SNI & de Jong Loneliness, CEL	Yes, No	X	Social Connection, Social Isolation, Loneliness	Structure, Function, Quality
I miss having people around me	de Jong Loneliness	Yes, No	X	Social Connection, Social Isolation, Loneliness	Structure, Function, Quality
I can find companionship when I want it	UCLA Loneliness	None of the Time, Some of the Time, Often	X	Loneliness	Function
I feel isolated from others	UCLA Loneliness	None of the Time, Some of the Time, Often	X	Loneliness	Function
I lack companionship	UCLA Loneliness	None of the Time, Some of the Time, Often	X	Loneliness	Function
I feel no one really knows me well	UCLA Loneliness	None of the Time, Some of the Time, Often	X	Loneliness	Function
In the past 2 weeks, I have participated in organizations such as: Social clubs, residents groups, or committees	DSSI & Steptoe Isolation	Yes, No	X	Social Connection, Social Isolation	Structure, Function, Quality
In the past 2 weeks, I have participated in organizations such as: Religious groups	DSSI & Steptoe Isolation	Yes, No	X	Social Connection, Social Isolation	Structure, Function, Quality
I feel useful to my family and friends (the people that are important to me)	DSSI	Yes, No		Social Connection	Structure, Function, Quality
There is someone available to me who shows me love and affection	Berkman-Syme SNI	Yes, No		Social Connection	Structure, Function
Other than today, in the past 3 days, I have been to other rooms of my home besides the room where I sleep	Life Space	Yes, No		Community Mobility	N/A
Other than today, in the past 3 days, I have been to places outside my home, within my town or community	Life Space	Yes, No		Community Mobility	N/A
I feel part of a group of friends	UCLA Loneliness	None of the Time, Some of the Time, Often		Loneliness	Function
There are people I feel close to	UCLA Loneliness	None of the Time, Some of the Time, Often		Loneliness	Function
There are people I can talk to	UCLA Loneliness	None of the Time, Some of the Time, Often		Loneliness	Function
I worry about being by myself	UCLA Loneliness	None of the Time, Some of the Time, Often		Loneliness	Function
I feel my interests and ideas are not shared by those around me	UCLA Loneliness	None of the Time, Some of the Time, Often		Loneliness	Function
In the past 2 weeks, I had little interest or pleasure in doing things	Patient Health Questionnaire (PHQ-2)	Not at All, Several Days, More than Half the Days, Nearly Every Day		Depression	N/A
In the past 2 weeks, I felt down, sad, or hopeless	PHQ-2	Not at All, Several Days, More than Half the Days, Nearly Every Day		Depression	N/A
I tend to bounce back after illness or hardship	Connor-Davidson Resilience	None of the Time, Some of the Time, Often		Resilience	N/A
In the past 6 months, I had an emotional loss (e.g., death of a family member or friend)	N/A	Yes, No		Adverse Life Event	N/A
In the past month, I had a negative change in my health	N/A	Yes, No		Adverse Life Event	N/A
When thinking about the people in my life, I have about this many: Children	Steptoe Isolation	None, One, Two or More		Social Isolation	Structure
When thinking about the people in my life, I have about this many: Other family members	Steptoe Isolation	None, One, Two or More		Social Isolation	Structure
When thinking about the people in my life, I have about this many: Friends	Steptoe Isolation	None, One, Two or More		Social Isolation	Structure
When thinking about the people in my life, I have about this many: People I feel close to and could call for help	Steptoe Isolation	None, One, Two or More		Social Isolation	Structure
In the past 2 weeks, I had contact (including face-to-face, telephone, or written/email/text message contact) with: My children	Steptoe Isolation	Yes, No		Social Isolation	Structure
In the past 2 weeks, I had contact (including face-to-face, telephone, or written/email/text message contact) with: Other family members	Steptoe Isolation	Yes, No		Social Isolation	Structure
In the past 2 weeks, I had contact (including face-to-face, telephone, or written/email/text message contact) with: My friends	Steptoe Isolation	Yes, No		Social Isolation	Structure
It is easy for me get to appointments, grocery stores, places of worship, and other locations	N/A	Yes, No		Community Mobility	N/A
My sense of belonging to my local community is…	Brief Sense of Community Scale	Very Strong, Somewhat Strong, Somewhat Weak, Weak		Community Cohesion	N/A

^*^Concepts and aspects of social connection measured found at (16).

### 2.3 Data collection

This study used a cross-sectional, internet-delivered questionnaire to collect data from older adults ages 60 years and older. Participants were recruited nationwide through a Qualtrics Internet Panel (34) from June 2019 to September 2019. Given the potential sampling bias introduced by convenience sampling, quota sampling parameters were employed to ensure diversity among participants in terms of age, sex, race, and geography (35). A total of 4,101 older adults completed the survey, of which 19 were omitted for missing data. The resulting analytic sample included 4,082 participants from all 50 states and two U.S. territories. All survey procedures were approved by the Texas A&M University Institutional Review Board (IRB2019-0375).

### 2.4 Statistical analyses

Data were analyzed using R in an exploratory fashion to generate a brief and practical scale to identify threats to social connectedness (i.e., risk for social disconnectedness) among older adults. Several methods were applied to assess the underlying dimensionality of the data and construct measures of different factors related to risk (i.e., limited opportunity for social interactions and lacking emotional fulfillment from social interactions). First, after assessing relevant data assumptions (e.g., local independence, monotonicity, item variance), a unidimensional item response theory (IRT) model was fit to confirm the item’s relationship with the measure of risk and correlate participant scores (i.e., θ_s_) on this measure. IRT guided item reduction from the initial 36 items to the resulting 13-item scale. Second, clustering techniques were used to assess the number of subgroups present in the sample based on participants’ response patterns to the instrument items. Third, a structural equation model (SEM) was constructed to confirm the relationship among the latent variables on this risk dimension measured by the 13-item scale.

#### 2.4.1 Item response theory assessment

Items were recoded to reflect a binary solution (risk/no risk). Two (2PL) and three (3PL) parameter logistic models (36, 37) were fit to both the initial 36 items and 13-item versions of the scales in which the primary dimension was assumed to be risk. S-χ^2^ was used to detect items that did not fit well within each model, respectively. Several items were flagged as ‘poorly’ performing items in that they did not discriminate among participants at any point on the primary dimension. These items were discarded through the iterative process of pruning. A one parameter logistic model (i.e., 1PL or Rasch Model) (38) was also fit, which does not allow for discrimination, to confirm the theory that the two parameter logistic model best fit these data. The item response model was recalibrated on the subset of items selected for inclusion into the 13-item scale on the basis of the original item parameters and domain knowledge of the researchers. Results from recalibrating item and participant parameters for the 13-item scale closely mirrored results for the initial 36-item set, suggesting minimal impact on measuring the primary dimension when using the 13-item subset of items. All IRT models were fit using the MIRT Package in R (39).

#### 2.4.2 Dimensionality assessment

An unsupervised neural network approach was used to explore the underlying dimensionality of the participants responding to the 13-item scale. Four different clustering algorithms were compared utilizing a k-fold method of selecting criterion. A “leave one out” k-fold methodology allows assessment of individual item importance to the stability of the clustering solutions. Furthermore, it allows multiple solution sets to be compared in a data-driven manner to assess a level of agreement on the underlying number of sub-groups present in the data. Partitions around medioids (PAM) (40, 41) was ultimately selected as the best option to cluster the data based on validation metrics (42–45). A two-cluster solution representing low-and high-risk participants was chosen by validating the cluster membership with comparisons of proportions of endorsement for items representing either low or high risk.

#### 2.4.3 Structural equation modeling (SEM)

A structural equation modeling framework was utilized to confirm the relationship among the latent factors governing the responses in the 13-item scale. Based on participant response patterns, three groups of items emerged, which were used as latent factors in the SEM. Clustering results gave evidence for items that were related to each other in response pattern among the participants, as well as being conceptually related. The three factors that emerged were related to the physical opportunity of the older adult to interact with others (i.e., Factor 2), their emotional fulfillment from these interactions (or lack thereof) (i.e., Factor 3), and general feelings of disconnectedness (i.e., Factor 1) captured by select broad items from the UCLA Loneliness Scale. The structure of the model was implemented to support the hypothesis that physical opportunities for interaction and the emotional fulfillment of interactions would predict how older adults perceived their general disconnectedness. As such, Factor 1 was treated as endogenous and regressed onto the two exogenous factors (i.e., Factors 2 and 3). While allowing the exogenous factors to correlate to emulate the potential interrelation between these factors in real-world conditions, no other individual item variances or error variances were allowed to correlate in the final model.

## 3 Results

Table 2 presents sample characteristics of the 4,082 older adults who participated in this study. On average, participants were age 69.58 (±5.24), and the majority was female (58.5%), non-Hispanic (84.7%), White or Caucasian (73.5%), and lived with a spouse or partner (57.1%). Approximately 47% of participants had a college degree or more, with 35.6% reporting some college or technical school education and 17.6% reporting a high school education or less. On average, participants reported 3.29 (±2.56) chronic conditions, with the most frequently reported conditions being hypertension (53.4%), high cholesterol (48.0%), arthritis or rheumatic disease (31.7%), chronic pain (23.0%), and diabetes (20.4%).

**Table 2:** Sample characteristics (*n* = 4,082).

Age (range 60 to 98)	69.58 (±5.24)
Sex	
Female	2,390 (58.5%)
Male	1,692 (41.5%)
Ethnicity	
Not Hispanic	3,458 (84.7%)
Hispanic	624 (15.3%)
Race	
White or Caucasian	2,999 (73.5%)
Black or African American	801 (19.6%)
Asian	46 (1.1%)
American Indian or Alaska Native	18 (0.4%)
Other Race or Multiple Races	218 (5.3%)
Education level	
High school or less	718 (17.6%)
Some college or technical school	1,452 (35.6%)
College graduate or more	1912 (46.8%)
Lives with spouse or partner	
No	1752 (42.9%)
Yes	2,330 (57.1%)
Number of chronic conditions (0–19)	3.29 (±2.56)

### 3.1 Item response theory assessment

Two and three parameter logistic IRT models were calibrated for the initial 36 items, and again for the 13-item screener in part on some results from the dimensionality assessment above alongside the long-form calibration. The items included were determined to cover a good nomeopathic span (46) regarding an older adult’s risk for limited social interactions in terms of physical opportunities to interact with others and the emotional fulfillment from social interactions.

As seen in Table 3, the overall model fit was better for the 13-item compared to the initial 36 items. The 2PL model was selected because the discrimination values for each of the items allowed for the selection of items that were relevant along the entirety of the trait continuum. The 1PL model was tested for its parsimonious nature, but it was not expected to fit as well as a 2PL model due to the discrimination parameter. Lastly, a 3PL variant was tested to account for random guessing, but the increase in parameterization did not outweigh the lack of statistical and conceptual fit to the task. This is most evident by the improvement of fit observed for each of the transitions from the 1PL to 2PL models illustrated in Table 3. In theory, there are items that ‘discriminate’ more on different components of the trait spectrum, and if this assumption is to be considered true, an increase in fit should be observed (as it is here). The drop of AIC/BIC and − 2 x LL for each model stops with the addition of the third parameter for each item. The guessing parameter does not afford any sizable increase in fit, and as such, the most appropriate form of a model for these data is the 2PL model (47, 48).

**Table 3:** Model fit comparison for 1PL, 2PL, and 3PL IRT solutions.

Model	-2 x LL	Change	Parameters	Change	AIC	BIC
*Initial 36 items*						
1PL	−56,695		36		113,464	113,698
2PL	−51,700	4,995	72	36	103,545	104,000
3PL	−51,695	5	108	36	103,607	104,289
*13-Item scale*						
1PL	−21,465		36		42,959	43,048
2PL	−20,383	1,082	72	36	40,819	40,984
3PL	−20,379	4	108	36	40,837	41,083

As seen in Table 4, the M2 model fit statistic (49) indicates that the 13-item scale of the test fits better than the initial 36 item for every model metric. More specifically, the relative fit for the 13-item scale improves over the initial 36 items as illustrated by the confidence interval for the RMSEA not containing 0.05 and the CFI larger than 0.95. It can be concluded that the 13-item variant reproduces data under the model more consistently, if not better than the 36-item scale, likely due to a loss of complexity by reducing ‘noise’ from modeling items that are not as strongly related to the trait of interest.

**Table 4:** 2PL Model fit comparisons for initial 36 items and 13-item scale.

				RMSEA				
				CI: 95%				
	M2	df	RMSEA	5%	95%	SRMSR	TLI	CFI
Initial 36 items	9793.87	594	0.0616	0.0605	0.0627	0.0648	0.891	0.896
13-Item scale	579.029	65	0.0440	0.0410	0.0473	0.0372	0.975	0.979

Individual item fit was calculated using a S-χ^2^ statistic (50, 51). This goodness-of-fit test shows how well the expected score during parameter estimation conforms with the observed score at different places on the trait continuum. Table 5 illustrates how individual item fit improved in many cases when reducing the scale from 36 to 13 items. In other cases, fit remained relative the same.

**Table 5:** Comparison of 2PL item fit statistics in the initial 36 items and 13-item scale.

	Difficulty		Discrimination		$S\chi^{2}$ $\left(p\;\left(S\chi^{2}\right)\right)$	
Items	Initial 36	13-Item	Initial 36	13-Item	Initial 36	13-Item
**Factor 1**						
I feel isolated from others	0.05	0.46	2.92	2.93	0.025(<0.001)	0.024(0.001)
I lack companionship	0.05	0.46	2.22	2.35	0.029(<0.001)	0.012(0.116)
I feel no one really knows me well	0.27	0.27	1.82	1.69	0.029(<0.001)	0.017(0.020)
**Factor 2**						
In the past 2 weeks, I have participated in organizations such as… Social clubs, residents groups, or committees	−2.48	−3.19	0.44	0.34	0.012(0.046)	0.010(0.175)
In the past 2 weeks, I have participated in organizations such as… Religious groups	−1.93	−2.42	0.40	0.31	0.005(0.356)	0.006(0.336)
I avoid socializing because it is hard to understand conversations, especially when there is background noise	2.77	3.22	0.87	0.72	0.011(0.067)	0.021(0.002)
I can find companionship when I want it	2.29	2.6	1.65	1.34	0.016(0.004)	0.016(0.026)
**Factor 3**						
I am satisfied with the relationships I have with my family	1.51	1.5	1.91	1.94	0.012(0.043)	0.021(0.002)
I am satisfied with the relationships I have with my friends	1.62	1.62	2.60	2.68	0.016(0.005)	0.000(0.726)
I have as much contact as I would like with people I feel close to and who I can trust and confide	1.28	1.22	2.11	2.41	0.020(<0.001)	0.016(0.026)
There are enough people I feel close to and could call for help	1.41	1.46	3.17	2.80	0.003(0.403)	0.011(0.153)
I am content with my friendships and relationships	1.25	1.21	3.35	4.14	0.021(<0.001)	0.010(0.191)
I miss having people around me	1.18	1.39	2.79	3.19	0.026(<0.001)	0.021(0.003)

Further evidence to support the fit of the model comes from the use of the participant parameters. When the binary responses from 13-item scale were summed to create a total score (range from 0 to 13) and correlated with the θ_s_ parameter generated from the IRT process, a strong and significant correlation was observed (*r* = 0.896, *p* < 0.001). This indicates that the summed score of binary responses can serve as a statistical proxy for an older adult, which lends itself to more practical use in research and practice settings. Additional information about practical scoring of the 13-item scale is provided elsewhere in this manuscript.

Reliability in the transition from the initial 36 items to the 13-item scale was assessed. The initial Cronbach’s alpha for the initial 36 items was 0.81. This reliability metric was reassessed after the item selection process was employed following the results of the IRT models dimensionality assessment, and SEM model. This transition revealed favorable results for a 13-item scale was seen as the Cronbach’s alpha for the 13-item scale was 0.80, which indicates no significant loss in reliability in the sample of 4,082 individuals after omitting 23 items. To assess any potential subgroup biases with the 13-item scale, reliability coefficients were calculated for variable categories by sex, ethnicity, race, education, living with partner, and reporting multi-morbidity. Each respective calculation yielded Cronbach’s alpha coefficients equal to or greater than 0.78, which indicates consistently strong internal reliability for subgroups within the current sample.

### 3.2 Dimensionality assessment

Three different measures were chosen to evaluate the number of clusters in the solution (i.e., older adults based on their responses to the 13 items). Connectiveness, similarity, and compactness were selected to compare clustering methodologies. Measures of connectivity and silhouette width should be minimized, and the Dunn index should be maximized (42). As such, it appears that the optimal number of clusters is 2. The Dunn index uses the ratios of the smallest distance between observations not in the same cluster to the largest intra-cluster distance; therefore, it has a bias towards a larger number of clusters.

The stability measures assessed the change in distance metrics used across clustering techniques when one column is removed from the dataset and the clustering technique is re-ran in a k-fold fashion. Therefore, the stability measure through this leave-one-out procedure should be able to detect if there are any clustering methods that are sensitive to a particular item. Average proportion of non-overlap (APN); average distance of means (ADM); and average distance (AD) between cases in the same clusters should all be minimized.

**Figure 1:**
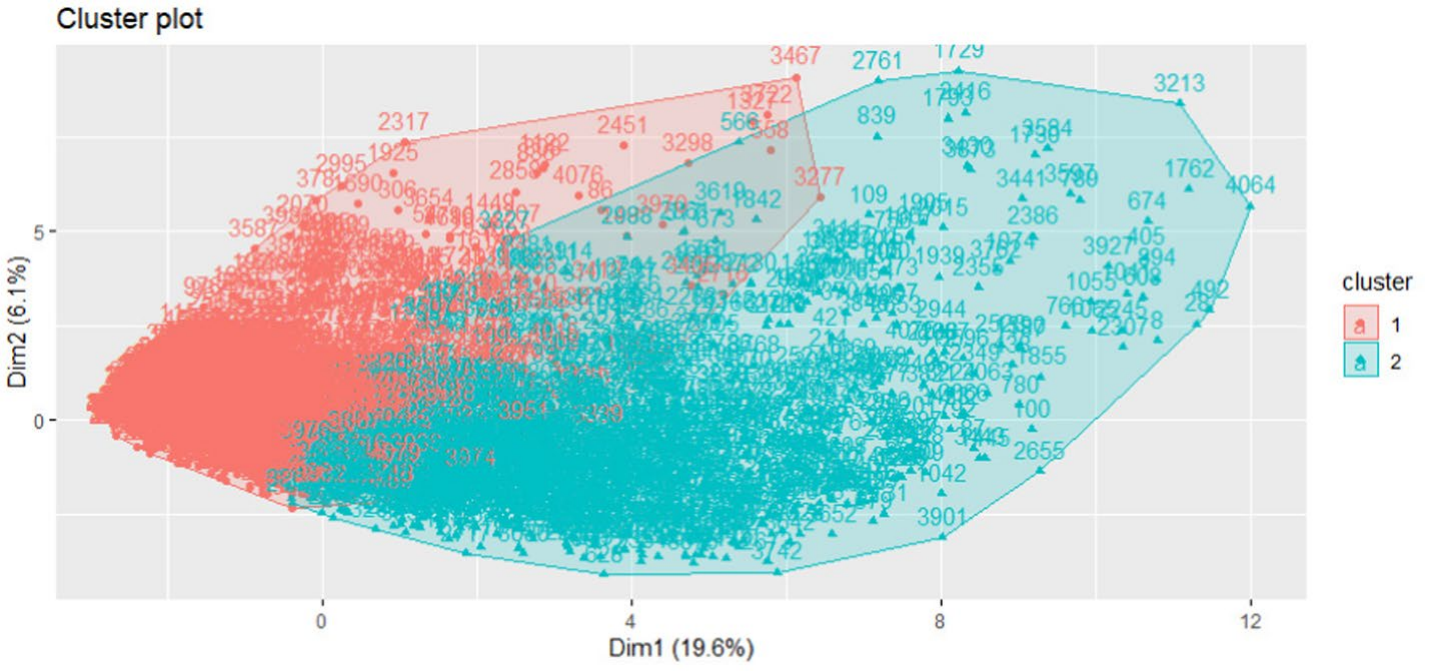
Plot of component scores for 2-cluster solution, scaled in 2 dimensions.

The separation of the clusters is one of the most important metrics to consider when the goal is to classify individuals in an applied setting. Plotting the component scores, as seen in Figure 1, illustrates that we can expect clear separation on the first dimension and subtle separation on the second vertical dimension for the clusters.

**Figure 2:**
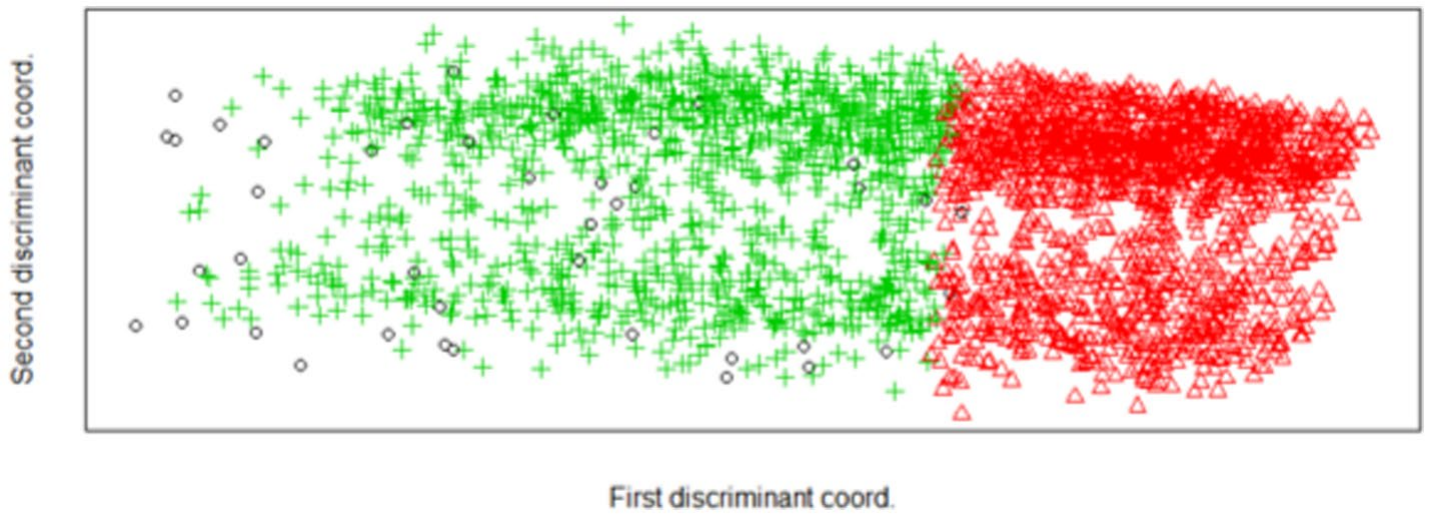
Plot of discriminant coordinate for a 2-cluster solution, scaled in 2 dimensions.

A discriminant clustering algorithm was applied to the data to ‘trim’ fringe cases by selecting a discriminant function, which maximizes the differences between these cases (52, 53). Figure 2 illustrates the results of these analyses and shows that when attempting to maximize differences between clusters with a discriminant function, more separation can be seen on the vertical dimension. It should be noted that the primary discriminant coordinate still categorized the older adults primarily despite clear vertical separation of the clusters.

### 3.3 Structural equation modeling (SEM)

**Figure 3:**
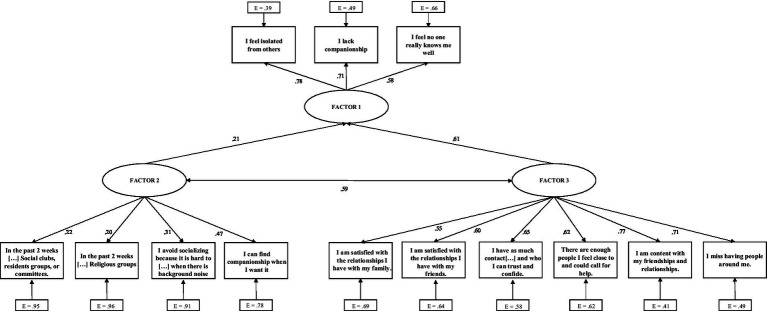
SEM relating physical opportunity and emotional fulfillment factors to general risk.

A structural equation modeling framework was leveraged against the data for the 13-item scale and fit using three latent factors (see Figure 3). Identification of items for each factors utilized results from the dimensionality assessment and IRT model parameters in conjunction with the intended ecological application of each item. The three items in Factor 1 (i.e., ‘I lack companionship’; ‘I feel isolated from others’; and ‘I feel like no one really knows me well’) were considered the target of the prediction because they were deemed to capture general feelings of disconnectedness and they were the strongest items indicative of risk. Meanwhile, the remaining items represent two distinct ways in which this outcome can occur, namely a physical opportunity component (Factor 2) and an emotional fulfillment component (Factor 3).

The model fits exceptionally well (RMSEA = 0.048; CFI = 0.954) (54, 55). The structural regression component favored the contribution of the Factor 3 items over the Factor 2 items (Coefficients of 1.348/0.832, respectively). However, the physical opportunity component significantly predicted the Factor 1 items. All coefficients were positive, indicating that higher scores on the individual items were directly related to an increase in the Factor 1 risk probability (i.e., general feelings of social disconnectedness). Covariance between Factor 2 and Factor 3 were permitted, which were only roughly related with a true correlation of 0.59. This suggests that the physical opportunity of an older adult to interact with others does not directly infer that the emotional fulfillment from of those interactions is positive.

## 4 Discussion

This study reports the process used to develop and validate the Upstream Social Interaction Risk Scale (U-SIRS), a 13-item scale to assess threats to social connectedness among older adults in terms of their social interactions. This scale is novel in that it was created from a modified composite of seven existing scales used to measure various aspects of social connectedness, and it aims to measure “upstream risk” by scoring scale items binarily to identify the maximum amount of risk. This scale is practical in that it emphasizes general threats to social connectedness, as well as elements associated with physical opportunities to socially interact and the emotional fulfillment from such interactions (or the lack thereof), which may enable a more actionable approach to connecting older adults to services, resources, and programs. The potential utility of this scale in clinical and community settings is vast because it may help identify a wider scope of risk using a single measure and may provide sites with a practical alternative to existing scales, which can alleviate the screening and data collection burden associated with using multiple scales simultaneously.

The process to develop this scale was two-fold in that it engaged a diverse set of clinicians, professionals, and community members to help define the possible universe of questions to measure threats to social connectedness. Through this process, these experts prioritized and omitted items that seemed overlapping and of limited practical value to identify risk. They suggested language changes to the items and response choices, which helped the items’ readability, comprehension, and feasibility for use in real-world circumstances. Further, the items were refined to be phrased in a more positive or uplifting manner in attempt to avoid evoking negative feelings simply from completing the items. Interestingly, when attempting to define the universe of possible items, no single existing scale was included in its entirety and most items were altered in terms of their phraseology or response choices. This process resulted in 36 items with strong face validity as a starting point for further testing and scale refinement.

After collecting data from over 4,000 older adults ages 60 years and older nationwide, the item response theory (IRT) process analyzed the initial 36 items to quantify the latent trait of this general risk or threat to social connectedness. Through this process, a subset of 13 items were identified as contributing most to the overall latent trait of risk and that the other 23 items did not provide much information to the latent trait. Further, the Cronbach’s alpha for the initial 36 items and the reduced set of 13 items were comparable and above 0.80, indicating strong internal consistency reliability. Taken together, the reduced 13-item scale was identified as the strongest set of items to measure this latent trait of risk.

After reducing the scale from 36 to 13 items, the dimensionality assessment examined potential patterns of participant responses for the 13-item scale. In these analyses, it was determined that the 2PL solution clearly stratified this older adult population into high and low risk groups. This reinforces that these 13 items can be utilized to create separation based on risk levels, which has practical implications for future studies to establish risk-based scoring for the scale (i.e., identifying cut-points for risk thresholds).

Among the most compelling findings of this validation process is that the SEM confirmed that the U-SIRS-13 scale consists of three distinct factors, which include general feelings of disconnectedness (identified by three items from the UCLA Loneliness Scale), physical opportunities for social interaction, and emotional fulfillment from social interactions (or the lack thereof). The SEM model indicates that the distinct physical opportunity and emotional fulfillment sub-scales roughly predict each other (*r* = 0.59), but both strongly predict the general feelings of social disconnectedness. This reinforces the notion that an older adult may have the physical opportunity to interact with others, but that physical opportunity will not always infer that they perceive emotional fulfillment from such opportunities. The SEM model also shows that each scale item does not function on each scale factor, respectively. For example, the item ‘I lack companionship’ informs only the general feelings of disconnectedness factor and functions solely on this dimension. This particular item does not inform the other two factors, except that it can be predicted by the other items in the scale (i.e., those from the physical opportunity and emotional fulfillment sub-scales). As such, it is important to stress that, despite the U-SIRS-13 comprising three distinct factors, the IRT shows that all 13 items conform to a singular trait, which is also confirmed by the strong internal reliability coefficient for the binarily scored data (i.e., Cronbach’s alpha=0.80). Therefore, it is recommended that the U-SIRS-13 be scored as a continuous count variable of risk rather than scoring each sub-scale independently. However, examining the risk for each item may help practitioners identify threats to structure, function, and/or quality aspects of social connection and make appropriate resources, programs, or services.

An important and practical finding of this study is that the theta score from the IRT strongly correlated (*r* = 0.896, *p* < 0.001) with the number of items that participants endorsed as ‘risk’ (i.e., a count variable of binarily-scored items ranging from 0 to 13). Therefore, the results from the theta parameters from IRT model validates the use of total count score in practice. It is recommended that future efforts deploy these items using a uniform set of response choices capable of identifying varying levels of risk for each item. Table 6 presents the recommended U-SIRS-13 items, response choices, and practical scoring for dichotomizing items to identify the maximum amount of risk (i.e., “upstream risk”). After dichotomizing responses for each item, the 13 items should be summed to create a count variable ranging from 0 to 13, with higher values indicating more risk (17, 18). Additional ongoing demonstration studies and evaluation efforts have utilized this practical scoring for the U-SIRS-13 to examine the scale’s internal reliability. As seen in Table 7, the internal reliability of data collected with the practically-scored U-SIRS-13 remains consistently strong (i.e., Cronbach’s alpha ranging from 0.78 to 0.85) for general samples of older adults as well as those purposively recruited for social engagement interventions. Future and ongoing studies will also examine alternative scoring strategies for the U-SIRS-13.

**Table 6:** Recommended practical scoring for U-SIRS-13.

U-SIRS-13 Items	Never	Sometimes	Often
I feel isolated from others	0	1	1
I lack companionship	0	1	1
I feel no one really knows me well	0	1	1
I can find companionship when I want it	1	1	0
In the past 2 weeks, how often have you attended: social clubs, residents’ groups, or committees	1	0	0
In the past 2 weeks, how often have you attended: religious groups	1	0	0
I avoid socializing because it is hard to understand conversations, especially when there is background noise	0	1	1
I am satisfied with the relationships I have with my family	1	1	0
I am satisfied with the relationships I have with my friends	1	1	0
I have enough contact with people I feel close to and who I can trust and confide	1	1	0
There are enough people I feel close to and could call for help	1	1	0
I am content with my friendships and relationships	1	1	0
I miss having people around me	0	1	1
Score items binarily, then sum to create a count variable from 0 to 13 (higher values indicate more risk)			

**Table 7:** Internal reliability of practically-scored U-SIRS-13 in ongoing demonstration studies and evaluation efforts.

Sample population	Sample size	Mean (±SD)*	Cronbach’s alpha
Current Study Sample: Nationwide cross-sectional survey of adults ages 60+ years	4,082	3.51 (±2.68)	0.80
Baseline data from a Texas-based intervention to improve the wellness of caregivers of people living with dementia	95	6.48 (±3.71)	0.85
Nationwide cross-sectional survey of full-time employees ages 18+ (only those ages 60+ included here)	419	5.58 (±3.59)	0.83
Baseline data from multi-state friendly calling/visiting intervention with older adult Meals on Wheels clients	303	6.99 (±3.29)	0.78
Baseline data from a multi-state Community of Practice evaluating tablet-and workshop-based interventions to help older adults get connected	404	5.51 (±3.81)	0.85
Baseline data from a Texas-based clinical intervention to address social disconnectedness among dual-eligible older adults using Community Health Workers and community navigation	453	7.88 (±3.51)	0.83
Sample of older adults ages 65+ years with varying levels of cognitive impairment	85	3.88 (±2.98)	0.78
Nationwide cross-sectional data from a federally-funded clearinghouse dedicated to identify risk for social disconnection and provide resources to help older adults get connected	174	8.57 (±3.18)	0.79
Cross-sectional assessment of older adults calling a California-based crisis hotline	44	7.02 (±3.84)	0.85

^*^U-SIRS-13 items scored binarily, then summed to create a count variable from 0 to 13.

### 4.1 Limitations

This validation study was not without limitation. Despite conducted with a large sample of diverse older adults ages 60 years and older across the United States (*n* = 4,082), probabilistic sampling was not used to select participants. Therefore, sociodemographics somewhat align with those of the greater older adult population in the United States (e.g., age, sex, ethnicity, living alone), these data were not nationally representative. Further, while Qualtrics panels are strong methods to recruit large samples quickly (35), the internet-based nature of recruitment and data collection may have introduced selection bias in terms of technology access, education level, and affluence (i.e., not reaching those with more potential risk for social disconnectedness). For these reasons, the analytic sample in this study may not be generalizable to the overall older adult population in the United States, especially among those with lower socioeconomic status. Data were self-reported, thus subject to social desirability bias, especially given the stigmatization of loneliness and social disconnection in the United States (56, 57). Despite these potential shortcomings, the strengths of this study include a diverse set of professionals who assisted in the initial item selection, a robust set of statistical analyses to generate and assess the U-SIRS-13, and the emerging evidence of replicability of the practically-scored scale.

### 4.2 Future research directions

Findings from this study highlight the need for additional research efforts to advance the utilization and application of the U-SIRS-13. First, beyond the set of ongoing demonstration studies and evaluation efforts, additional replication studies are needed among diverse samples of older adults. More specifically, studies are needed to examine the appropriateness and reliability of the scale among samples of older adults with varying races and ethnicities, sexual orientations, socioeconomic statuses, impairments (e.g., sensory, mobility, cognitive), disabilities, and other known threats to social disconnectedness (e.g., partner status, living alone, limited transportation, caregiver status, multi-morbidity, fall history, food insecurity). Additional studies should also be performed to identify the utility and statistical integrity of this scale for younger adults. Second, statistical efforts are needed to identify risk-related thresholds and establish cut-points for risk levels. While higher scores on the U-SIRS-13 are indicative of higher risk, the identification of cut-points may help researchers and practitioners utilize the scale to identify high-risk older adults and make informed decisions for programs and services. Third, as another form of validation, efforts are needed to identify the concordance between professional perceptions of social disconnectedness relative to self-reported threats of disconnectedness using the U-SIRS-13 among older adults in clinical and community settings. Such efforts may provide insights into the ability of those serving older adults to recognize various threats to social connection and the relative advantage of administering the U-SIRS-13. Fourth, although the U-SIRS-13 is considered brief, professionals in clinical and community settings may find it lengthy for use during intake and routine assessments. As such, future research should consider validating a reduced set of items indicative of probable risk and requiring further assessment (e.g., akin to administering the 2-item PHQ as a “first step” approach, then administering the full 9-item PHQ if risk on the 2-item PHQ is identified) (58). Fifth, while anticipated to be sensitive to change over time, the U-SIRS-13 should be used in concurrent validity assessments to predict other outcomes as well as an outcome evaluation in relevant interventions to assess its ability to identify baseline risk and improvement post-intervention.

## 5 Conclusion

This study documented the development and validation of the Upstream Social Interaction Risk Scale (U-SIRS-13), a 13-item scale to document threats to social connection, which was created using a compilation of items from seven previously validated scales. The U-SIRS-13 contains an interrelated set of three distinct sub-scales that measure feelings of general disconnectedness, physical opportunities for social interactions, and emotional fulfillment from social interactions (or lack thereof). Despite the distinct sub-scales, the IRT and SEM support its use as a single scale. The strong correlation between the theta score and summed composite of binarily-scored items supports the practical utilization of the practically-scored U-SIRS-13 in research and practice settings (further supported by emerging replicability in demonstration studies and evaluation efforts). Building upon the legacy of existing scales, especially the UCLA Loneliness Scale, the U-SIRS-13 adds to the existing inventory of measures about social connection (16) with a more encompassing and sensitive scale that can identify “upstream risk” and detect the maximum amount of threat to social connectedness among older adults. Future efforts are needed to examine and identify risk levels and thresholds, which can help those using the U-SIRS-13 to classify older adults’ risk level, determine whether their risk is in terms of physical opportunities to interact or emotional fulfillment from interactions, and refer older adults to appropriate programs and services.

## Data Availability

The raw data supporting the conclusions of this article will be made available by the authors, without undue reservation.
